# Perleche, a New Parasitic Disease of Children

**Published:** 1886-09

**Authors:** F. R. Campbell


### *PERLECHE, A NEW PARASITIC DISEASE OF CHILDREN.*

By F. R. CAMPBELL, M. D.

Translated from *Le Progrès Medical.*

There exists among the children attending the schools in the provinces, a mild disease, the parasitic nature of which has just been demonstrated by Dr. Austin Lemaistre, Prof in the Medical School of Limoges. It will not be uninteresting to our readers to publish the facts given by our distinguished confrere concerning this new disease, which is not mentioned in any treatise on medicine. In the Limousin district, where the disease is quite common, the inhabitants designate it by the name of *perleche* (French, *perlecher*, to lick), because of the burning sensation which leads the children to lick their lips. It is also called *Bridou*, because the commissures of the lips are, as it were, fastened together, (*bridèes*), the disease first making its appearance at the angles of the mouth, and extending from that locality. The epithelium first assumes a whiteish hue, then becomes soft, and is easily detached; the disease then attacks the cutaneous surface about the mouth, and becomes at once manifest. As a rule only the superficial layers of the epidermis are affected and the true skin is not diseased. Sometimes there are slight fissures in the commissural folds which cause considerable pain and some hemorrhage when the child opens his mouth. The lesions bear a marked resemblance to the mucous patches and fissures of syphilitic children.

The duration of the disease is not ordinarily greater than from fifteen days to a month. Some children are attacked nearly every year, and relapses are not at all rare. Such is the clinical appearance of the disease of which Lemaistre is the god-father To complete the clinical history we will state that the affection is not of a grave character, and is unattended by constitutional disturbances; but it causes pain on movement of the mouth and is unsightly. As Lemaistre has demonstrated beyond question, the malady is contagious and is propagated by the inconvenient drinking vessels commonly used in provincial schools, all the pupils drinking from the same cup without cleansing either it or their mouths as is customary in polite society.

Having had occasion to make microscopic examinations of the secretions from the lips of a number of children affected with perleche, Dr. Lemaistre constantly found micro-organisms and soon came to the conclusion that the disease was of a parasitic nature. In preparations colored with methylene violet, he ascertained the presence of numerous globular bacteria and diplococci. In places the bacteria formed isolated masses and colonies of from 20 to 300 individuals. Their favorite place of growth is the border of epithelial cells; often the cells are invaded and destroyed by the bacteria. Lemaistre concluded from his first studies that perleche is characterized by the presence of a microbe belonging to the class schizomycetes and group sphero-bacteria, micrococci.

Culture experiments were then made. Five Pasteur tubes were filled with sterilized bouillon. These were inoculated by taking platinum wires, after being heated to a white heat, cooled, placed in contact with the perleche of five children, and then dipped into the bouillon of the tubes which was kept at the temperature of the body, ninety-eight degrees. At the end of ten hours numerous whitish flakes were observed in the tubes. Under the microscope there were seen isolated sphero-bacteria and others arranged in the form of a figure eight with movements identical with those found in the morbid secretions. Long chaplets or streptococci were also seen. The flocculi were made up of an interlacement of innumerable chains, whence the name *streptococcus plicatalis* which the author gives to the microbe. The most astonishing phenomenon observed by Lemaistre in his studies was the rapidity with which the micro-organisms developed; within a few hours they would increase to incalculable numbers. In all the tubes the same microbe was always found, without the admixture of any other variety. Pursuing his investigations further, Lemaistre endeavored to find the microbes of perleche in the drinking water of certain schools where the disease was prevalent. A child from L'Ecole des Feuillants, affected with the disease, lived in the suburbs and drank water from a fountain in a neighboring meadow. Perleche is very common in the neighborhood. A woman, whose children had recovered from the disease on leaving this district, states that they were reinfected immediately upon their return. Provided with these facts, Lemaistre collected with a sterilized pipe the last drop of water in a basin of water from the fountain. Microscopic examination failed to reveal the streptococcus. Twenty Pasteur tubes, containing sterilized bouillion were inoculated with the water. After being kept ten hours at a temperature of eighty-six degrees, the bouillon became turbid and in thirty hours flocculi were seen, bacteria and diplococci also appeared, but there was no evidence of mature streptococci. In order to detect among the numerous colonies of bacteria in the tubes, the microbe which he sought, Lemaistre used the jelly of *fucus crispus* prepared according to Miquels method and spread upon Bristol board. Then the boards were plunged into the bouillon inoculated from the water and placed in sterilized tubes stopped with cotton. After the lapse of some days numerous distinct colonies were developed on the boards. Some of these consisted of diplococci, which, placed in bouillon developed the streptococcus plicatalis. It was then known that the microbe of perleche is identical with that found in certain potable waters and that the disease is produced by the use of such water. The explanation of the origin and propagation of the disease is quite plausible. The micrococci of perleche are found in infected wells, fountains, and in stagnant water. When this water is drawn into pails and pitchers and placed in the warmer air of the house or kitchen, the micrococci develop and assume the form of chains. The wooden pails of schools are always moist and seldom washed, thus becoming a suitable culture ground for the germs; from these other persons, other utensils, and even the well or spring itself may be infected. The microbe is found only at the angles of the mouth because it is anaerobic, and finds here sufficient moisture and shelter for its development. But even this shelter is imperfect and it is for this reason that the microbe is limited in its action and soon dies, thus accounting for the mildness of the disease. The following are some of the statistics of the disease collected by Lemaistre: Of 5,500 children, who attend the primary schools of Limoges, 312, about 1-17, were attacked with the disease. Some schools did not have a single case, in others nearly all were affected. The latter schools were in the suburbs, where the water was supplied from wells and springs. These statistics were collected in the winter, when the disease is at its lowest ebb. In the spring and summer the malady increases to a considerable extent. Although the writer's experience is confined to Limoges, the disease prevails in many other localities. In the village of Perigord, a few months ago, the majority of the children were affected with perleche.

With regard to treatment, we will state that it is exceedingly simple. Touching the affected integument with sulphate of copper or alum is very efficacious. Boric acid is useless and Lemaistre assures us that the streptococcus plicatalis will develop rapidly in borated solutions. Treatment is, however, of no great importance, as the majority of cases will recover without any applications whatever provided ordinary cleanliness is observed. As the microbes develop in the pails and tin cups, it is of the utmost importance to see that these are properly cleansed and disinfected. Boiling water or dry heat will serve as valuable disinfectants for the vessels, but infected wells and springs must be abandoned.

Finally, the medical inspection of schools should correct the traditionally unclean habits of the school-room, which propagate perleche and a host of other parasitic affections of childhood. There is thus a matter of school hygiene, besides the scientific importance of the discovery of Dr. Lemaistre, which reflects upon him the greatest honor. We have but one criticism to offer to the work of Dr. Lemaistre. After isolating and cultivating the infectious microbe of perleche he should have reproduced the disease by means of inoculation. The disease is benignant and the cure easy, and it is surprising that this experiment has been omitted in M Lemaistre's remarkable work.

